# Appendiceal Diverticulitis Clinically Masquerading as an Appendiceal Carcinoma

**DOI:** 10.1155/2014/837860

**Published:** 2014-12-03

**Authors:** Tadashi Terada

**Affiliations:** Department of Pathology, Shizuoka City Shimizu Hospital, Miyakami 1231, Shimizu-Ku, Shizuoka 424-8636, Japan

## Abstract

Appendiceal diverticulosis is a rare condition. Herein reported is a case of appendiceal diverticulosis and diverticulitis clinically masquerading as appendiceal carcinoma. A 62-year-old woman presented with abdominal pain. US and CT showed a tumor measuring 5 × 4 × 4 cm in vermiform appendix. Colon endoscopy showed mucosal elevation and irregularity in the orifice of vermiform appendix. A biopsy of the appendiceal mucosa showed no significant changes. Clinical diagnosis was appendiceal carcinoma and wide excision of terminal ileum, appendix, cecum, and ascending colon was performed. Grossly, the appendix showed a tumor measuring 5 × 3 × 4 cm. The appendiceal lumen was opened, and the appendiceal mucosa was elevated and irregular. The periappendiceal tissue showed thickening. Microscopically, the lesion was multiple appendiceal diverticula. The diverticula were penetrating the muscle layer. The mucosa showed erosions in places. Much fibrosis, abscess formations, and lymphocytic infiltration were seen in the subserosa. Abscesses were also seen in the diverticular lumens. Some diverticula penetrated into the subserosa. The pathologic diagnosis was appendiceal diverticulitis. When they encounter an appendiceal mass, clinicians should consider appendiceal diverticulitis as a differential diagnosis.

## 1. Introduction

The diverticular disease of vermiform appendix is a rare condition [1, 2]. It may cause inflammation (diverticulitis) and rupture and is associated with pseudomyxoma peritonei, malignant tumors, and other complications [3]. However, appendiceal diverticulitis clinically masquerading as appendiceal carcinoma is very rare.

## 2. Case Report

**Figure 1:**
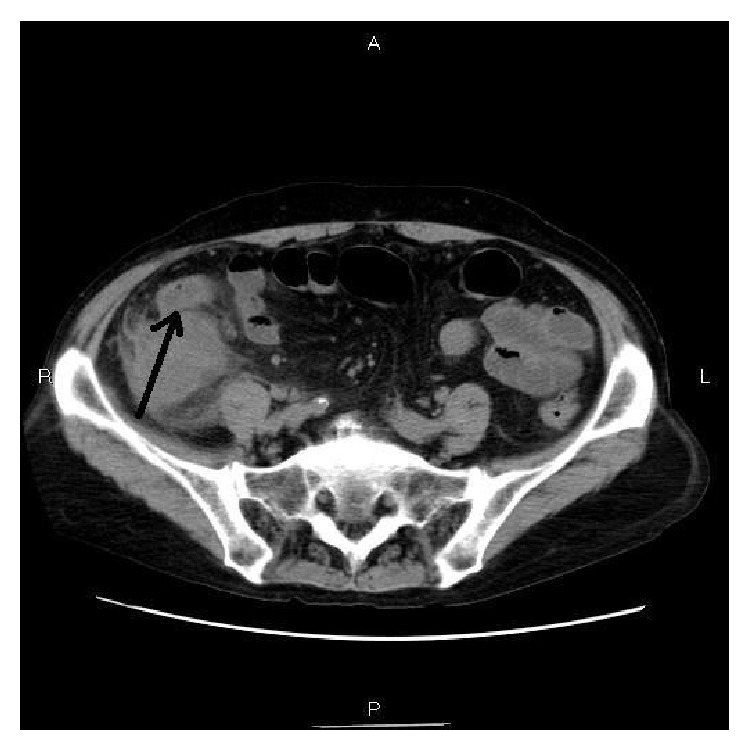
Abdominal CT. The appendix shows a mass measuring 5 × 4 × 4 cm. The appendix cannot be identified (arrow).

**Figure 2:**
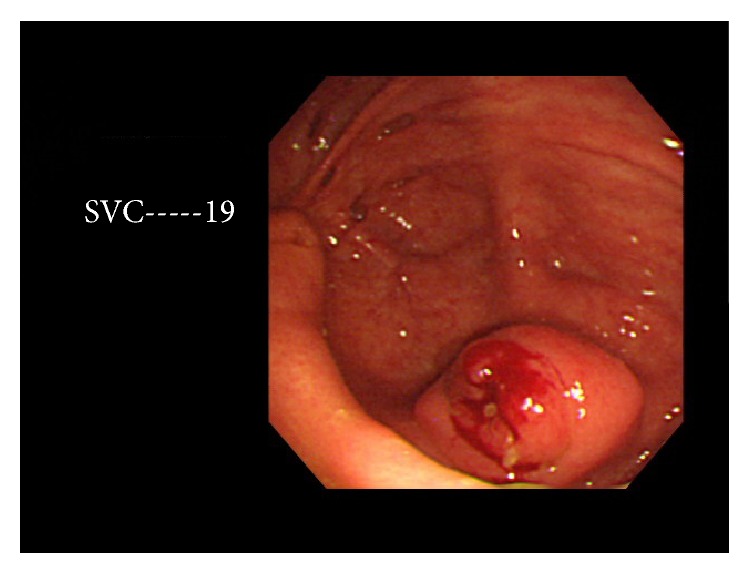
Colonoscopic findings at the level of cecum. Appendiceal orifice is seen. It shows mucosal erosions and irregularities.

A 62-year-old Japanese woman with HCV-related cirrhosis and small hepatocellular carcinoma presented with abdominal pain. The small hepatocellular carcinoma had been treated with microwave coagulation therapy and cured. A blood laboratory test revealed elevation of cirrhosis-related enzymes, bilirubin, and AFP. No leukocytosis or increased C-reactive protein was present. CA125 was elevated. US and CT showed a tumor measuring 5 × 4 × 4 cm in the vermiform appendix (Figure 1). Colon endoscopy showed mucosal elevation and irregularity in the orifice of vermiform appendix (Figure 2). A biopsy of the appendiceal mucosa showed no significant changes. Clinical diagnosis was appendiceal carcinoma and wide excision of the terminal ileum, appendix, cecum, and ascending colon was performed.

**Figure 3:**
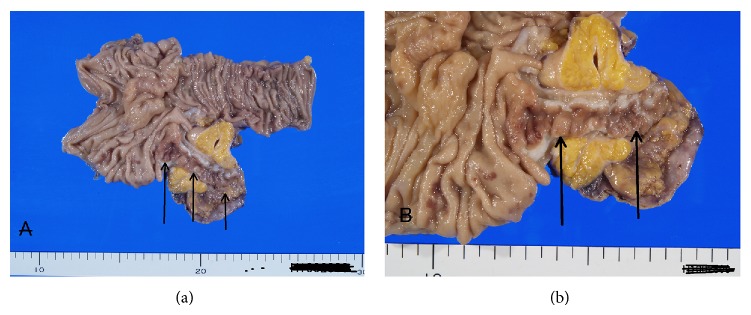
Gross features of the appendiceal tumor. (a) The appendix (arrow) is open. (b) High power view of the appendix. The appendiceal mucosa (arrows) shows irregularity.

**Figure 4:**
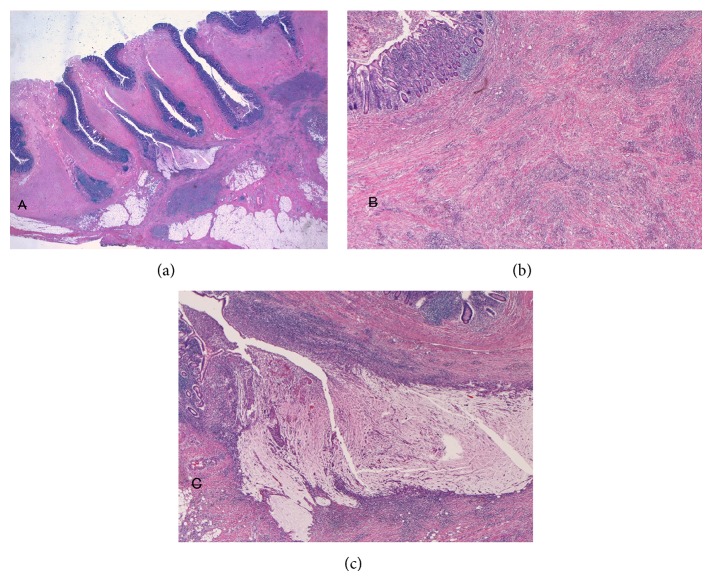
(a) Low power features of appendiceal diverticula. Many diverticula are seen in this section. The diverticula penetrate the muscular layer, suggesting that they are congenital in origin. Much fibrosis and inflammation are seen in the subserosal walls which contain a very thin muscle layer. HE ×5. (b) High power view shows luminal abscess and much fibrosis and lymphocytic infiltration of the subserosa. HE ×50. (c) High power view shows diverticula with penetration. HE ×50.

Grossly, the appendix shows a tumor measuring 5 × 3 × 4 cm (Figure 3(a)). The appendiceal lumen was open (Figure 3(a)), but the mucosa was elevated and irregular (Figure 3(b)). The periappendiceal tissue showed thickening (Figures 3(a) and 3(b)). Microscopically, the lesion was multiple appendiceal diverticula (Figure 4(a)). The diverticula were penetrating the muscle layer (Figure 4(a)). The mucosa showed erosions in places. Much fibrosis, abscess formations, and lymphocytic infiltration were seen in the subserosa (Figure 4(b)). Abscesses were also seen in the diverticular lumens. Some diverticula penetrated into the subserosa (Figure 4(c)). The pathologic diagnosis was appendiceal diverticulitis.

## 3. Discussion

The appendiceal diverticulum has been classified into congenital and acquired ones [1, 2]. The former has complete appendiceal walls, while the latter lacks muscular walls. The present case appears congenital in origin. About half of patients with appendiceal diverticulosis are asymptomatic, but it can cause diverticulitis, abscess formation, rupture, perforation, and hemorrhage and is associated with pseudomyxoma peritonei and malignant transformation [1, 2]. The incidence of appendiceal diverticulosis ranges from 0.27% to 3.56% in surgical cases [2, 4] and from 0.20% to 1.99% in autopsy series [4, 5]. In recent years, the incidence has been reported in 1.7% (22/1361) [6], 1.7% (10/575) [7], and 2% (67/3343) [8] of surgical cases. Only about 15% of appendiceal diverticula develop diverticulitis [1]. Therefore, appendiceal diverticulitis is very rare disease condition.

It is interesting that the present case was clinically diagnosed as appendiceal carcinoma. In a review of the English literature, the author could not find cases of appendiceal diverticulitis clinically masquerading as appendiceal carcinoma. The lack of blood inflammatory changes and positive mass formation in the imaging modalities of appendix led to the erroneous clinical diagnosis of appendiceal carcinoma in the current case. Therefore, it must be stressed that clinically appendiceal tumor may be due to appendiceal diverticulosis or diverticulitis. In recent years, relationship between appendiceal diverticulosis and appendiceal tumors or tumor-like conditions has been advocated.

Dupre et al. [6] described that appendiceal diverticulosis is associated with appendiceal carcinoids, adenoma, mucinous tumors, and adenocarcinoma. Lamps et al. [9] described that appendiceal diverticulosis is associated with low grade mucinous neoplasms and pseudomyxoma peritonei. The causal relationship between appendiceal diverticula and tumor formation is unclear, but it may be possible that the presence of tumors causes increased luminal pressure of the appendix, thus giving rise to the development of diverticulosis. It is also possible that the presence of appendiceal diverticulosis may cause tumor formation. This issue remains to be elucidated.

In summary, when they encounter an appendiceal mass, clinicians should consider appendiceal diverticulitis as a differential diagnosis.
